# Preventive effect of ulinastatin on postoperative cognitive dysfunction through modulation of the gut microbiome: evidence from short-chain fatty acids

**DOI:** 10.1016/j.bbih.2026.101327

**Published:** 2026-08-12

**Authors:** Eun-Hwa Cho, Seung-Wan Hong, Eun-Hye Seo, Seong-Hyop Kim

**Affiliations:** a Department of Infection and Immunology, Konkuk University School of Medicine, Seoul, Republic of Korea; b Department of Anesthesiology and Pain Medicine, Konkuk University Medical Center, Konkuk University School of Medicine, Seoul, Republic of Korea; c Korea MRNA Vaccine Initiative, Gachon University, Songdo, Republic of Korea; d Department of Medicine, Institute of Biomedical Science and Technology, Konkuk University School of Medicine, Seoul, Republic of Korea; e Department of Medical Education, Konkuk University School of Medicine, Seoul, Republic of Korea

**Keywords:** Gut, Dysbiosis, Ulinastatin, Urinary trypsin inhibitor, Microbiome, Postoperative cognitive dysfunction

## Abstract

**Background:**

Increasing evidence indicates that gut dysbiosis and gut–brain axis dysfunction contribute to postoperative cognitive dysfunction (POCD) following general anaesthesia. Although ulinastatin, a urinary trypsin inhibitor, has been reported to exert anti-inflammatory and neuroprotective effects, its role in the gut microbiome and microbiome-related metabolites in POCD remains unclear.

**Methods:**

A total of 30 18-month-old male Sprague–Dawley rats were randomly allocated to the Ulinastatin group or the Control group. Ulinastatin (50,000 U/mL) or normal saline (1 mL) was intraperitoneally administered before general anaesthesia, which was maintained with isoflurane 1.5 vol% for 2 h. Cognitive function was assessed using the Y-maze test. *Lactobacillus* viable counts, short-chain fatty acid (SCFA) levels and nuclear factor erythroid 2-related factor 2 (Nrf2) expression were evaluated by microbiological analysis, enzyme-linked immunosorbent assay (ELISA) and immunohistochemical staining, respectively.

**Results:**

Y-maze performance did not differ between groups before general anaesthesia. However, a significant decline after anaesthesia was observed only in the Control group (*p* < 0.001). *Lactobacillus* viable counts showed a similar pattern, with a significant reduction in the Control group but preservation in the Ulinastatin group (*p* < 0.001). The Ulinastatin group had significantly higher SCFA levels in the gut, blood, and brain than did the Control group (all *p* < 0.001). Nrf2 expression in both the gut and brain was also significantly higher in the Ulinastatin group (both *p* = 0.0001).

**Conclusion:**

Preoperative administration of ulinastatin prevented cognitive decline after general anaesthesia. The protective effect of ulinastatin was associated with control of gut dysbiosis.

## Highlights

• Microbiome therapy reduced neuroinflammation and positively affected cognitive function, though findings varied.
• Pre anaesthesia, ulinastatin administration had a preventive effect on cognitive decline, consistent with past reports.
• Ulinastatin prevented cognitive decline, preserved gut Lactobacillus, and altered SCFAs and Nrf2, suggesting neuroprotection.

## 1 Introduction

Postoperative cognitive dysfunction (POCD) is a common neurological complication following surgery, particularly among older patients (Liu et al., 2022a), and is characterized by impairments in memory, attention, executive function and information processing speed (Arefayne et al., 2023; Zhao et al., 2024). These cognitive disturbances may persist after hospital discharge, and negatively affect functional recovery and quality of life.

Although the mechanisms underlying POCD remain incompletely understood, neuroinflammation plays a key role in its development (Li et al., 2022; Yang et al., 2022b). Recently, neuroinflammation in POCD has been reported to be associated with dysbiosis in the gut (Wang et al., 2021b). Dysbiosis in the gut is defines as a condition of disruption of microbial diversity, leading to increasing potentially harmful microbiomes and decreasing beneficial microbiomes in the gut (Winter and Bäumler, 2023). Several attempts to control gut dysbiosis have shown improvement in neuroinflammation (Bairamian et al., 2022; Baizabal-Carvallo and Alonso-Juarez, 2020; Wang et al., 2021b). Emerging evidence suggests that modulation of gut dysbiosis and associated changes in microbiome-related metabolites are associated with reduced microglial activation and altered microglial polarization in the hippocampus during neuroinflammation (Erny et al., 2015; Johnson et al., 2019; Tang et al., 2023). Accordingly, Sugita et al. concluded that microbiome-targeted therapy decreased inflammation and appeared to have a positive effect on cognitive function, although the findings did not reach consensus (Sugita et al., 2023). Considering this evidence, agents that show preventive effects on neuroinflammation in POCD (Cho et al., 2024a, 2024b) might be associated with control of gut dysbiosis.

Ulinastatin, a urinary trypsin inhibitor, is purified from fresh healthy urine (Lv et al., 2026). It has anti-inflammatory effect, and protective effect against tissue injury via suppression of inflammatory signaling pathways such as nuclear factor kappa-light-chain-enhancer of activated B cells (NF-κB) pathway inhibition and mitogen-activated protein kinase (MAPK) pathway modulation, reduction of oxidative stress, stabilization of vascular permeability, and modulation of coagulation process (Wang et al., 2021a; Yin et al., 2025). Therefore, it has been widely used for the treatment of inflammatory diseases, although it is available only in China, India, Japan, and Korea (Hey-Hadavi et al., 2023; Tong et al., 2024). Previous studies have also demonstrated that preoperative administration of ulinastatin has beneficial effects on postoperative cognitive decline in not only animal studies (Cho et al., 2024a, 2024b; Kim et al., 2023c) but also clinical situations (Duan et al., 2021; Liang et al., 2021). Considering the preventive effect of ulinastatin on cognitive decline via its own anti-inflammatory property and protective property against tissue injury, it might protect gut dysbiosis in occurrence of POCD. However, no study has investigated the effect of ulinastatin on gut dysbiosis in the context of POCD.

We hypothesised that the preventive effect of ulinastatin on POCD might be associated with control of gut dysbiosis. This study was designed to evaluate whether the preventive effect of ulinastatin on POCD was associated with control of gut dysbiosis.

## 2 Materials and methods

All experiments were conducted in accordance with the National Institutes of Health Guide for the Care and Use of Laboratory Animals. This study was designed, conducted and reported in accordance with the ARRIVE Guidelines 2.0. After approval by the Institutional Animal Care and Use Committee (IACUC) of Konkuk University (approval number: KU24046, KU26028), all experiments were conducted at the Konkuk University Laboratory Animal Research Center in accordance with IACUC guidelines.

### 2.1 Animal preparation

Eighteen-month-old male Sprague–Dawley rats were purchased from Koatech (Pyeongtaek, Korea). The rats were housed in cages with free access to food and water. The room was maintained on a standard 12-h light–dark cycle (lights on at 7:00 and lights off at 19:00) at a temperature of 25°C. The rats were acclimated to the experimental conditions for 7 days before the study and fed a standard diet *ad libitum*, with free access to water.

### 2.2 Behaviour test

#### 2.2.1 Schedule and apparatus

Behaviour test was performed just as the previous studies (Cho et al., 2024a, 2024b). After acclimation, the Y-maze test was performed to evaluate cognitive function on the day before general anaesthesia and 2 days after general anaesthesia. The Y-maze consisted of three arms (A, B, and C). Each arm was 50 cm long, 25 cm high, and 10 cm wide, and was connected to the other arms at an angle of 120°.

#### 2.2.2 Testing protocol

Before the start of the test, all arms were wiped with 70% alcohol, and arm C was blocked with a board at the center of the equilateral triangle to prevent access to arm C. The rat was placed at the end of arm A and allowed to adapt to the apparatus for 15 min, moving freely between arms A and B. After this 15-min adaptation period, the rat was returned to its cage and rested for 1 h. After the 1-h rest, all arms were wiped again with 70% alcohol, the board blocking arm C was removed to allow free access to all arms, and the rat was placed again at the end of arm A. Movement was recorded with a video camera for 5 min.

#### 2.2.3 Quantification and exclusion criteria

The total number of entries into each arm and spontaneous alternations were recorded, and the alternation ratio was calculated using the formula [(number of spontaneous alternations)/(total arm entries − 2)] × 100% (Cho et al., 2024a, 2024b; Li et al., 2020). Rats showing an alternation ratio of < 40% on the day before general anaesthesia were regarded as having pre-existing cognitive impairment and were excluded from the study (Kim et al., 2023b).

### 2.3 Animal grouping and general anaesthesia

#### 2.3.1 Animal grouping and drug administration

Grouping and general anaesthesia were performed just as the previous studies (Cho et al., 2024a, 2024b; Piao et al., 2022; Seo et al., 2023). The animals were randomly allocated to the Control group or the Ulinastatin group. The Control group received 1 mL normal saline and the Ulinastatin group received 1 mL ulinastatin (50,000 U; Ulistin®, Hanlim Pharm., Korea), administered intraperitoneally.

#### 2.3.2 General anaesthesia and mechanical ventilation

After administration, anaesthesia was induced with intraperitoneal ketamine 100 mg/kg (Yuhan, Korea) and xylazine 10 mg/kg (Sigma-Aldrich, USA). A heating pad was placed on the surgical platform to maintain the body temperature at approximately 37°C during anaesthesia. The rat was secured in the supine position and fastened on the surgical platform for endotracheal intubation using a 16 G catheter (45 mm; Dukwoo Medical, Korea). Correct placement of the catheter was confirmed by symmetrical chest expansion. The catheter was connected to a ventilator (Harvard Apparatus, USA). The ventilator settings were as follows: fraction of inspired oxygen, 0.5; inspired flow rate, 150 mL/min; tidal volume, 6 mL/kg; respiratory rate, 50 breaths/min; inspiration:expiration ratio, 1:1; and positive end-expiratory pressure, 5 cmH_2_O. Anaesthesia was maintained with isoflurane 1.5 vol% for 2 h, and the ventilator settings were maintained throughout. After 2 h of isoflurane maintenance, the isoflurane vaporiser was stopped. Mechanical ventilation was continued until full recovery of spontaneous ventilation was confirmed. After confirming recovery of spontaneous ventilation, the endotracheal catheter was removed and the rat was transferred to its cage.

### 2.4 Microbiome analysis using lactobacillus viable count

#### 2.4.1 Fecal sample collection and storage

Dysbiosis was evaluated using *Lactobacillus* viable counts. Faecal samples were collected to assess the gut microbiome on the day before general anaesthesia and 2 days after general anaesthesia. Samples were placed in 1-mL microtubes and stored at −20°C.

#### 2.4.2 Serial dilution and plating

For analysis, the stored samples were thawed. After thawing, 1 g of faeces was mixed with 9 mL normal saline in a 15-mL conical tube and thoroughly homogenised. The mixture was then serially diluted from 10^2^ to 10^7^ in conical tubes containing 0.9% normal saline. After dilution, 100 μL of each dilution was spread onto de Man–Rogosa–Sharpe agar plates (Microgen, Germany) using a spreader (SPL, Korea).

#### 2.4.3 Incubation and colony quantification

The plates were placed in a HEPA class 100 CO_2_ incubator (Thermo, USA) at 37°C with 5% CO_2_ and cultured for 24 h. After incubation, the plates were examined and colonies were counted. The viable count was expressed as log_10_ colony-forming units (CFU) per gram of faeces.

### 2.5 Tissue collection

Samples were obtained in the following order: blood, brain, and gut.

#### 2.5.1 Animal sacrifice under anaesthesia

After the Y-maze test 2 days after general anaesthesia, the rat was anaesthetised with isoflurane 5 vol% with oxygen 0.3 L/min and nitrous oxide 0.7 L/min in an induction chamber (Cho et al., 2024a, 2024b). After confirmation of adequate anaesthesia, the animal was sacrificed.

#### 2.5.2 Blood sample processing

The abdomen was opened to expose the abdominal aorta. A blood sample was obtained from the abdominal aorta using a 1-mL syringe and stored in an ethylenediaminetetraacetic acid tube (BD Vacutainer®, USA). The tube was then centrifuged at 1500 × *g* for 20 min at 4°C to separate the plasma. The separated plasma was transferred to a 5-mL conical tube and stored at −20°C.

#### 2.5.3 Brain tissue isolation and preparation

After blood collection, 1× phosphate-buffered saline (PBS) was perfused through the abdominal aorta until complete exsanguination was achieved and the liver colour changed from red to pale. Using scissors and forceps, the skull was opened and the brain was removed. The brain was divided into two hemispheres. The right hemisphere was prepared to evaluate short-chain fatty acids (SCFAs) by enzyme-linked immunosorbent assay (ELISA) and nuclear factor erythroid 2-related factor 2 (Nrf2) expression by Western blot. It was transferred to an Eppendorf® tube (Eppendorf, Germany) and stored at −20°C. The left hemisphere was prepared for assessment of Nrf2 expression by immunohistochemical staining and stored in a 15-mL conical tube containing 4% paraformaldehyde (PFA) (BIOSESANG, Korea) at 4°C for immunofluorescence analysis. This approach was adopted to avoid potential interference arising from repeated processing of the same tissue and to enable complementary analyses from the same animal, consistent with our previous studies (Cho et al., 2024a, 2024b). Because all interventions in this study were systemic rather than hemisphere-specific, systematic differences between the left and right hemispheres were not expected.

#### 2.5.4 Gut tissue isolation and preparation

After brain collection, the colon was removed from the gut. It was opened longitudinally and divided evenly into two parts. One portion was prepared for analysis of SCFAs by ELISA and Nrf2 expression by Western blot, transferred to an Eppendorf® tube, and stored at −20°C. The other portion was prepared for assessment of Nrf2 expression by immunohistochemical staining and stored in a 15-mL conical tube containing 4% PFA at 4°C for immunofluorescence staining.

### 2.6 SCFAs from gut, blood, and brain

The stored colon samples were thawed at room temperature and homogenised in 1× PBS (100 mg/mL). The homogenised tissue was transferred to a microtube and centrifuged at 12,000 × *g* for 15 min at 4°C (Cho et al., 2024a, 2024b). After centrifugation, the supernatant was collected into a microtube and analysed using an ELISA kit (MyBioSource, Canada). A microplate reader, reflecting combined SCFA levels rather than individual metabolites (acetate, propionate and butyrate), was used to measure SCFA concentrations.

The stored plasma was thawed at room temperature, transferred from the conical tube to a microtube, and analysed using the same method as for the colon samples. The stored right hemisphere was also analysed using the same procedure.

### 2.7 Immunofluorescence staining and Western blot for Nrf2 in gut and brain

Paraffin-embedded colon and brain sections were subjected to immunofluorescence staining. Sections were incubated with an anti-Nrf2 primary antibody (Invitrogen, USA) followed by an Alexa Fluor 488-conjugated anti-rabbit secondary antibody (Invitrogen, USA), and nuclei were counterstained with DAPI (4′,6-diamidino-2-phenylindole). Fluorescence images were acquired using a fluorescence microscope (Olympus, Japan). Detailed procedures are described in the Supplementary Materials.

Proteins were extracted from colon and brain tissues, and protein concentrations were determined using a bicinchoninic acid protein assay kit (Thermo, USA). Equal amounts of protein were separated by sodium dodecyl sulphate–polyacrylamide gel electrophoresis (SDS-PAGE), transferred onto polyvinylidene difluoride membranes, and incubated with an anti-Nrf2 primary antibody followed by a horseradish peroxidase-conjugated goat anti-rabbit IgG (H + L) secondary antibody (GenDEPOT, USA). Protein bands were visualized using enhanced chemiluminescence and the iBright CL1000 imaging system (Thermo, USA). Detailed procedures are described in the Supplementary Materials.

## 3 Statistics

### 3.1 Sample size determination from a pilot study

Because there is no report for the expression of *Lactobacillus* at the occurrence of POCD, the pilot study was conducted to determine sample size. On the hypothesis, the expression of *Lactobacillus* in the faeces 2 days after general anaesthesia, as the primary outcome, and SCFA concentrations and Nrf2 expression in the gut and brain as the secondary outcomes, respectively were determined. In the pilot study, which included 3 rats in the Control group and 3 in the Ulinastatin group, faecal *Lactobacillus* expression 2 days after general anaesthesia was 4.17 ± 1.94 log_10_ CFU in the Control group and 10.00 ± 6.29 log_10_ CFU in the Ulinastatin group. The SCFA concentration was 0.37 ± 0.40 pg/mL (gut) and 0.55 ± 0.46 pg/mL (brain) in the Control group and 8.29 ± 2.02 pg/mL (gut) and 10.99 ± 3.01 pg/mL (brain) in the Ulinastatin group. Nrf2 expression was 1.82% ± 0.46% (gut) and 1.63% ± 0.15% (brain) in the Control group and 8.05% ± 1.00% (gut) and 8.27% ± 1.37% (brain) in the Ulinastatin group. Based on the results of the pilot study, sample size determination was performed, using G*Power version 3.1.9.7 (Heinrich Heine University Düsseldorf, Düsseldorf, Germany). The effect size for the primary outcome was estimated as Cohen's d = 1.25. A total sample size of 30 for the primary outcome, as well as 4 samples each for SCFAs (gut and brain) and 6 samples each for Nrf2 (gut and brain) as secondary outcomes, was calculated to achieve a power of 0.9 with an α value of 0.05.

### 3.2 Statistical analysis

Statistical analyses and graphical presentations were performed, using GraphPad Prism software version 8.0.1.244 (GraphPad Software, La Jolla, CA, USA). For outcomes measured before and after general anaesthesia, including the Y-maze alternation ratio and faecal *Lactobacillus* count, a two-way repeated-measures analysis of variance (ANOVA) with time and group as factors was used to assess the time × group interaction. Planned comparisons were performed to compare values before and after general anaesthesia within each group using paired two-tailed t-tests and to compare values between the Control and the Ulinastatin groups at each time point using unpaired two-tailed t-tests. For outcomes measured once after general anaesthesia, including SCFA concentrations in the gut, blood and brain, and Nrf2 expression in the gut and brain between-group comparisons were performed using unpaired two-tailed t-tests. For all unpaired two-tailed t-tests, Welch's correction was applied when the assumption of equal variances was not met. Data are presented as mean ± standard deviation and *p* values < 0.05 were considered statistically significant.

## 4 Results

Thirty rats were enrolled and equally allocated to each group. There were no dropouts.

### 4.1 Behavioral test

**Fig. 1:**
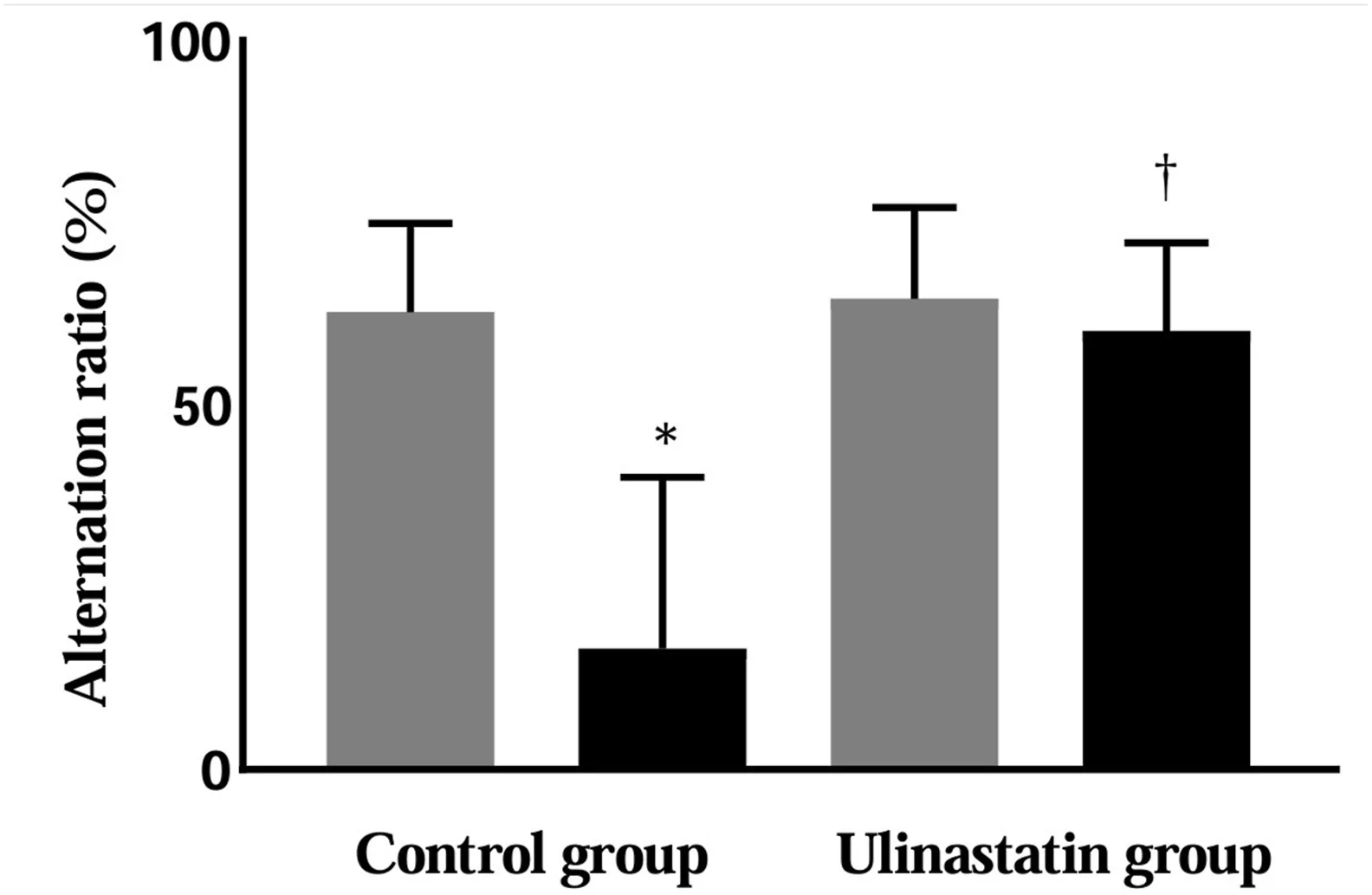
Cognitive function, using Y-maze test, on the day before general anaesthesia (represented with gray bar) and 2 days after general anaesthesia (represented with black bar). *: *p* < 0.001, compared with the day before general anaesthesia (represented with gray bar) and two days after general anaesthesia (represented with black bar) in Control group. ^†^: *p* < 0.001, compared with Control group.

The Y-maze alternation rate on the day before general anaesthesia did not differ between groups (*p* = 0.677). Repeated-measures ANOVA showed a significant main effect of time [F (1,28) = 32.408, *p* < 0.001] and a significant time × group interaction [F (1,28) = 29.283, *p* < 0.001], indicating that the change over time differed between groups (Fig. 1). In planned within-group comparisons, the alternation rate significantly decreased in the Control group (64.60% ± 17.11% → 43.56% ± 8.60%, *p* < 0.001), whereas no significant change was observed in the Ulinastatin group (67.27% ± 17.01% → 66.73% ± 15.58%, *p* = 0.345). On postoperative day 2, the alternation rate was significantly higher in the Ulinastatin group than in the Control group (Welch's *t*-test, *p* = 0.001) (Fig. 1).

### 4.2 Microbiome analysis using lactobacillus viable count

**Fig. 2:**
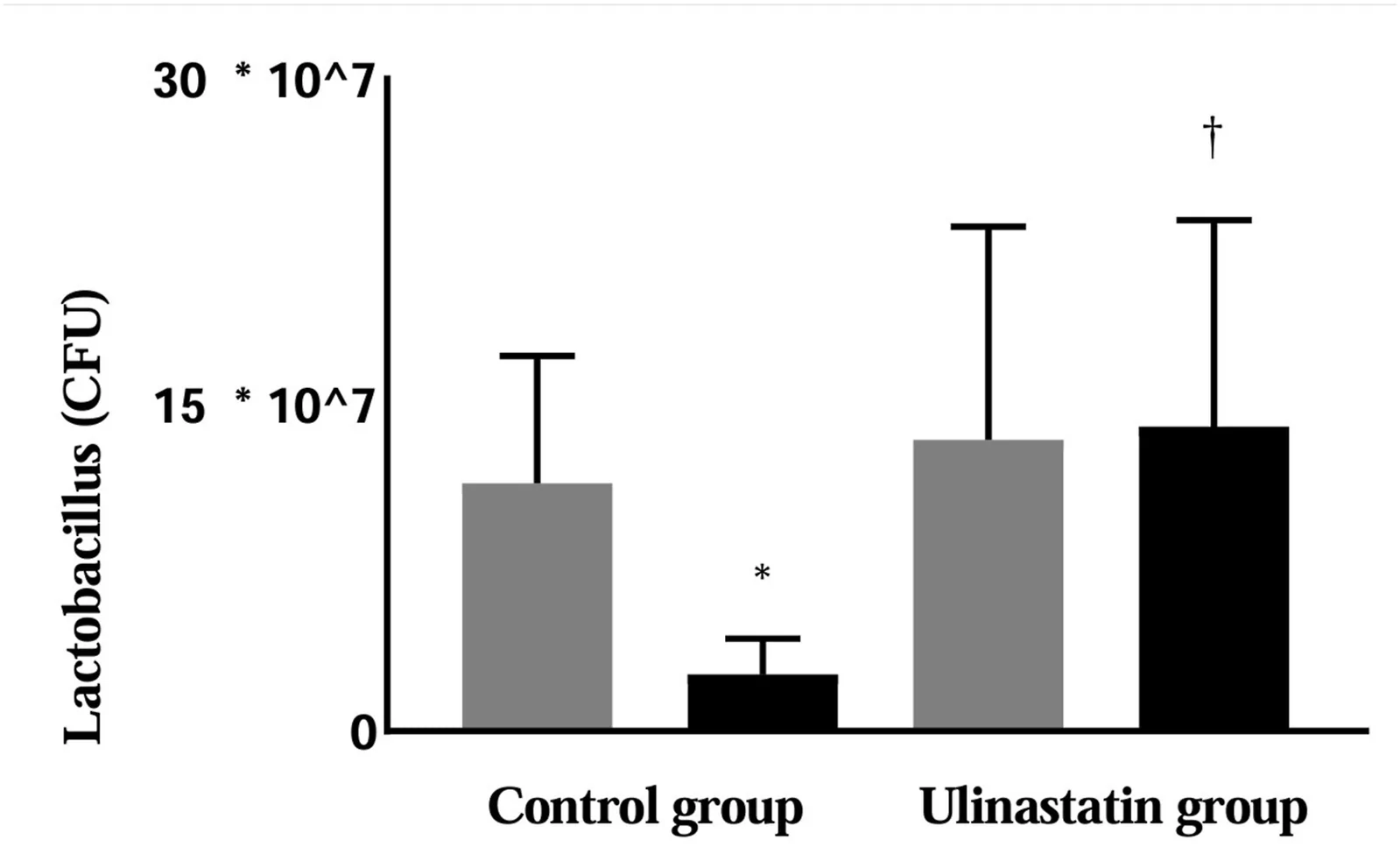
*Lactobacillus* viable count The expression of *Lactobacillus* on the day before general anaesthesia (represented with gray bar) and 2 days after general anaesthesia (represented with black bar). *: *p* < 0.001, compared with the day before general anaesthesia (represented with gray bar) and two days after general anaesthesia (represented with black bar) in Control group. ^†^: *p* < 0.001, compared with Control group. Abbreviations: CFU, Colony-forming unit.

Baseline faecal *Lactobacillus* counts did not differ between groups (*p* = 0.324). Repeated-measures ANOVA demonstrated a significant main effect of time (F (1,28) = 45.201, *p* < 0.001) and a significant time × group interaction (F (1,28) = 31.476, *p* < 0.001). In planned within-group comparisons, *Lactobacillus* counts significantly decreased in the Control group (11.47 ± 5.24 → 2.60 ± 1.30 log_10_ CFU/g, *p* < 0.001), whereas no significant change was observed in the Ulinastatin group (13.60 ± 6.34 → 12.80 ± 7.03 log_10_ CFU/g, *p* = 0.276). On postoperative day 2, *Lactobacillus* counts were significantly higher in the Ulinastatin group than in the Control group (Welch's *t*-test, *p* < 0.001) (Fig. 2).

### 4.3 SCFAs

**Fig. 3:**
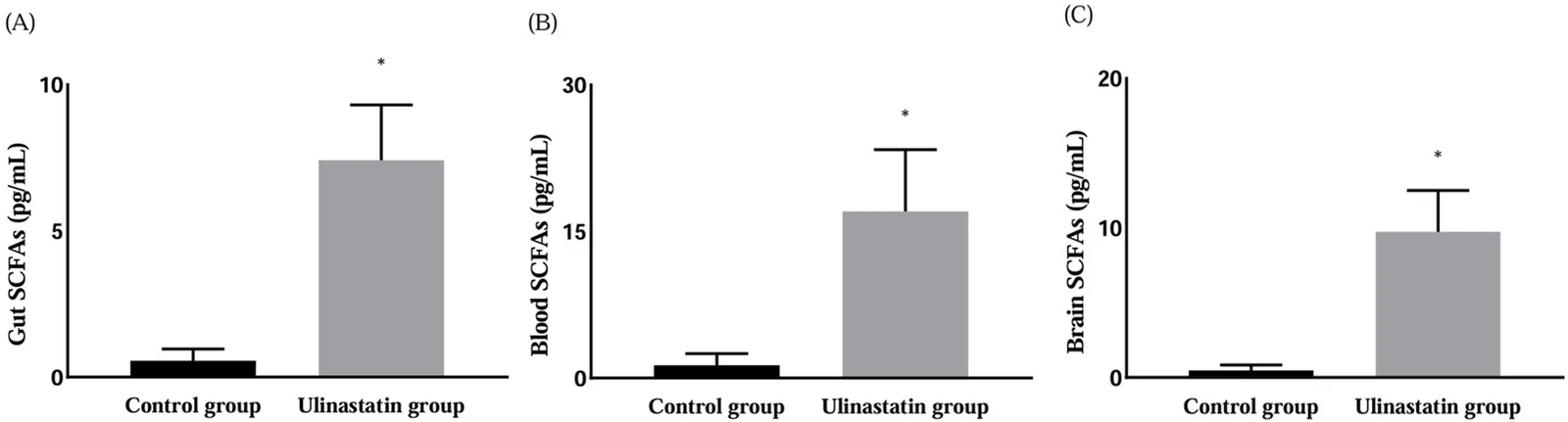
The expression of short-chain fatty acids (SCFAs). (A) Gut, (B) Blood and (C) Brain. *: *p* < 0.001, compared with Control group. **Abbreviations:** SCFAs, short-chain fatty acids.

The Ulinastatin group showed significantly higher SCFA levels than the Control group in the gut, blood, and brain (gut: 7.42 ± 1.89 *vs.* 0.57 ± 0.39 pg/mL, blood: 17.05 ± 6.33 *vs.* 1.32 ± 1.20 pg/mL, brain: 9.75 ± 2.75 *vs.* 0.47 ± 0.37 pg/mL; all *p* < 0.001) (Fig. 3).

### 4.4 Immunofluorescence staining and western blot for Nrf2

#### 4.4.1 Immunofluorescence staining and western blot for Nrf2 in gut

**Fig. 4:**
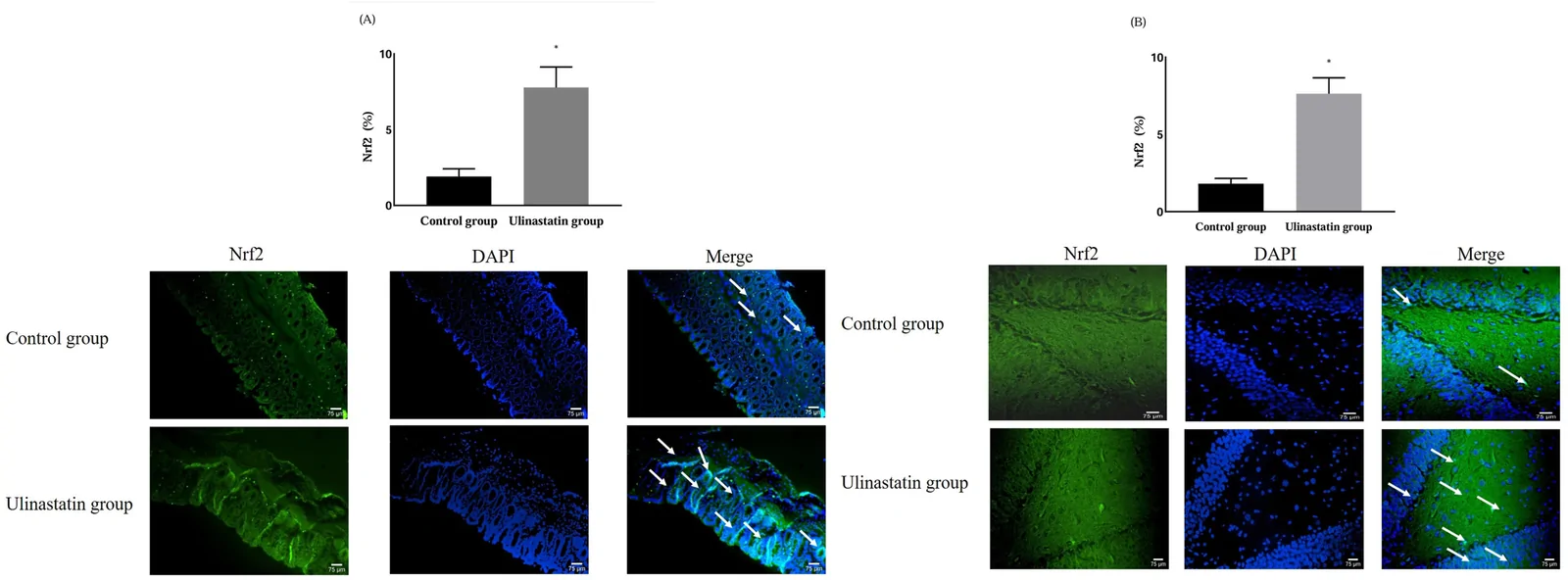
Immunofluorescence staining to detect nuclear factor erythroid-2-related factor 2 (Nrf2) (represented with white arrow). (A) Gut and (B) Brain. *: *p* = 0.0001, compared with Control group. **Abbreviations:** Nrf2, nuclear factor erythroid-2-related factor 2; DAPI, 4′,6-diamidino-2-phenylindole.

**Fig. 5:**
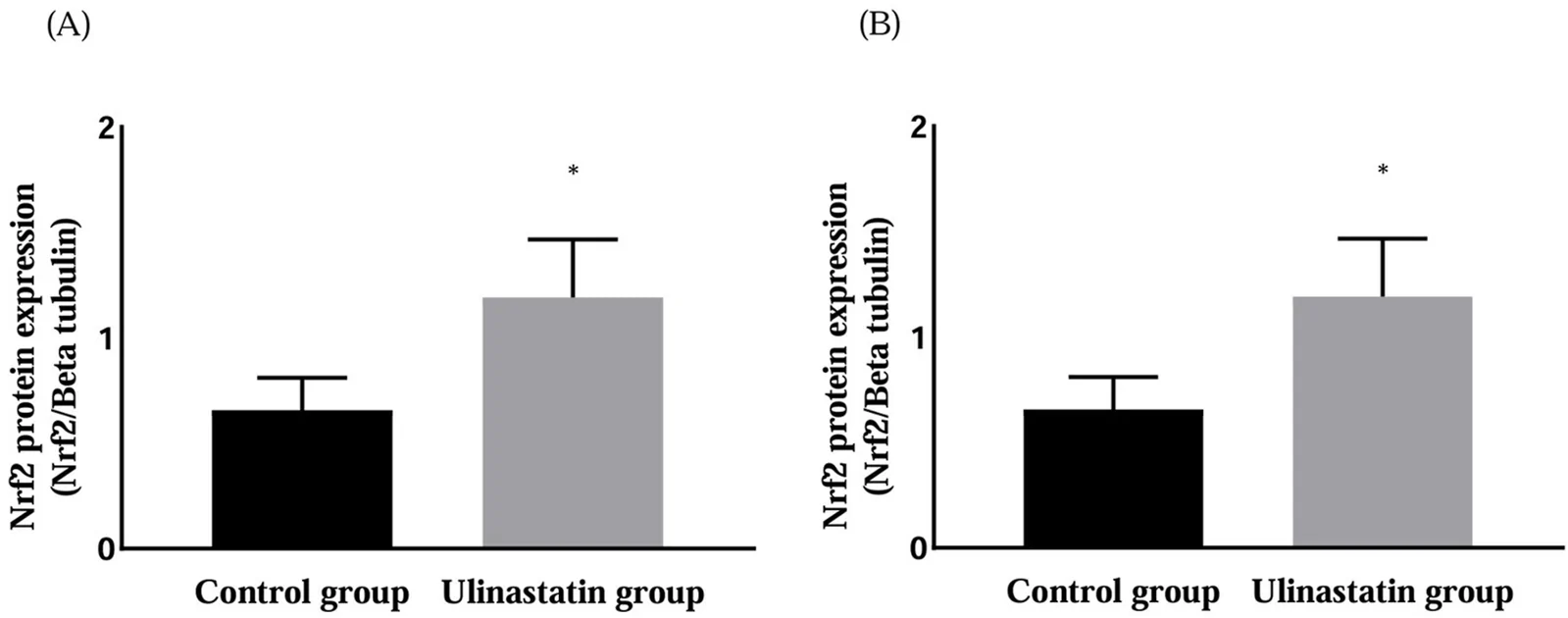
Western blot for quantification of nuclear factor erythroid-2-related factor 2 (Nrf2). (A) Gut and (B) Brain. *: *p* < 0.05, compared with Control group. **Abbreviations:** Nrf2, nuclear factor erythroid-2-related factor 2.

Nrf2 expression assessed by immunohistochemistry was significantly higher in the Ulinastatin group than in the Control group in gut and (7.65% ± 1.02% *vs.* 1.81% ± 0.34%, *p* = 0.0001) (Fig. 4-A). Nrf2 expression assessed by Western blot was also significantly higher in the Ulinastatin group (1.04 ± 0.05 *vs.* 0.78 ± 0.08, *p* = 0.008) (Fig. 5-A).

#### 4.4.2 Immunofluorescence staining and western blot for Nrf2 in brain

Nrf2 expression assessed by immunohistochemistry was significantly higher in the Ulinastatin group than in the Control group in brain (7.79% ± 1.35% *vs.* 1.92% ± 0.50%, *p* = 0.0001) (Fig. 4-B). Nrf2 expression assessed by Western blot was also significantly higher in the Ulinastatin group (1.19 ± 0.28 *vs.* 0.66 ± 0.15, *p* = 0.013) (Fig. 5-B).

## 5 Discussion

### 5.1 Summary of main findings

The present study showed that ulinastatin administration before general anaesthesia had a preventive effect on cognitive decline after anaesthesia, consistent with previous reports (Cho et al., 2024a, 2024b; Kim et al., 2023c). Ulinastatin also prevented the decline of *Lactobacillus* in the gut following general anaesthesia. This effect was supported by corresponding changes in SCFAs in the gut, blood, and brain and by changes in Nrf2 expression in the gut and brain.

### 5.2 The role of lactobacillus in neurologic disorders

We focused on *Lactobacillus* to explore the association between POCD and gut dysbiosis. *Lactobacillus* is a genus of Gram-positive, rod-shaped bacteria and constitutes a significant proportion of the microbiome in humans and animals. It is commonly referred to as acidophilus and is widely used as a probiotic in foods such as yoghurt and kimchi (Michael et al., 2020; Sauer and Han, 2021). Numerous studies have shown that *Lactobacillus* reduces inflammation in the central nervous system (CNS) and peripheral nervous system (Chen et al., 2022; Qin et al., 2022). Supplementation with *Lactobacillus* has antioxidant and neuroprotective effects (Ahmad Alwi et al., 2023; Kim et al., 2023a), and has been reported to improve or prevent pathological conditions, including neurological disorders (Gao et al., 2022; Oroojzadeh et al., 2022). It has also been shown to improve memory and learning by influencing neurotrophic factors (Aghamohammad et al., 2023) and to exert neuroprotective effects in traumatic brain injury (Ma et al., 2019). Meng et al. reported that dietary *Lactobacillus* regulated T-cell expression; reduced IL-1, IL-6, and tumour necrosis factor-α; and improved neural condition via modulation of intestinal status in neuritis (Meng et al., 2023). Moreover, *Lactobacillus* plays a key role in maintaining microecological balance through various mechanisms (Fan et al., 2021; Qin et al., 2022). These findings may underlie the preventive effect of ulinastatin on *Lactobacillus* decline and subsequent cognitive impairment observed in this study.

### 5.3 Gut-brain axis & SCFAs as metabolites from microbiomes in the gut

The gut–brain axis is defined as a bidirectional communication network between the intestine and the nervous system (Liu et al., 2025). This implies that the intestine can regulate nervous system function through multiple mechanisms (Liu et al., 2022b). Two conditions are necessary to support the gut–brain axis concept: metabolites from the microbiome must be identified in the gut, and these metabolites must be detected in the blood and brain after crossing the blood–brain barrier (BBB). To examine this in relation to *Lactobacillus*, we focused on SCFAs, which are produced in the gut as metabolites of *Lactobacillus* (Wang et al., 2021a). SCFAs are absorbed mainly in the caecum and colon (Martin-Gallausiaux et al., 2021) and can cross the BBB via monocarboxylate transporters in endothelial cells (Fock and Parnova, 2023). They contribute to maintenance of tight junction integrity and modulate inflammatory processes through inhibition of NF-κB and activation of Nrf2. SCFAs therefore influence BBB integrity and regulate CNS cells both morphologically and functionally, contributing to homeostasis (Dong and Cui, 2022; Silva et al., 2020). They have also been associated with effects on emotion, cognition, and the pathophysiology of CNS disorders (Cheng et al., 2024; Dong and Cui, 2022; Silva et al., 2020). The significantly increased SCFA levels observed in the Ulinastatin group across gut, blood, and brain may reflect preservation of *Lactobacillus* and provide evidence supporting the gut–brain axis.

### 5.4 Nrf2 as a regulator of SCFAs production

Nrf2 expression was evaluated by Western blot and immunohistochemistry as a marker of inflammatory regulation. Nrf2 plays a critical role in anti-inflammatory and antioxidant processes, regulating pro-inflammatory and anti-inflammatory cytokines and controlling gene expression via antioxidant response elements. Nrf2 signaling is also important for maintaining BBB tight junctions, and SCFA-induced activation of Nrf2 protects the BBB (Fock and Parnova, 2023). The major SCFAs produced in the human gut—acetate, propionate, and butyrate—are also regulated by Nrf2 (González-Bosch et al., 2021). Decreased Nrf2 expression is associated with the development and progression of neurodegenerative diseases (George et al., 2022). Conversely, activation of Nrf2 has been shown to slow progression of disorders such as Alzheimer's disease and Parkinson's disease by enhancing antioxidant defences and suppressing neuroinflammation (Qu et al., 2020; Yang et al., 2022a). Liang et al. reported that downregulation of Nrf2 in the brain contributed to the occurrence of POCD through neuroinflammation and oxidative stress (Li et al., 2023). In the present study, higher Nrf2 expression in the gut and brain after ulinastatin administration may be associated with increased SCFA levels and may further support the involvement of the gut–brain axis.

### 5.5 Study limitations

Several limitations should be considered in the presents study. First, Long-term storage of plasma and tissue samples is generally recommended at −80°C, particularly for periods exceeding 3 months (Xu and Kasprzyk-Hordern, 2023). However, we stored plasma and tissue sample at −20°C and analysed within 1 month. Previous studies have reported that short-term storage at −20°C has minimal effects on the stability and integrity of plasma and tissue samples (Flores et al., 2020). Therefore, the influence of the temperature on the results in the present study might be limited. Second, comprehensive analysis, such as 16 S ribosomal ribonucleic acid sequencing and metagenomics rather than assessment of a single microbiome in the gut, is required to evaluate overall gut dysbiosis. Although the present study has the strength that *Lactobacillus* was quantified as CFU, which reflects viability and functional status rather than composition profiling, the analysis should be interpreted as a targeted assessment of *Lactobacillus*-related changes rather than a comprehensive evaluation of gut dysbiosis. Third, although SCFAs were evaluated as representative microbial metabolites potentially linked to *Lactobacillus* in the present study, they are not exclusively produced by *Lactobacillus* and may originate from multiple gut microbial taxa (Ibrahim et al., 2024; Tang et al., 2023). The aim of the present study was not to establish a direct causal relationship between *Lactobacillus* and SCFA production, but rather to evaluate SCFAs as a functional readout of gut microbial metabolic activity potentially involved in the gut–brain axis. Moreover, combined SCFA levels rather than individual metabolites (acetate, propionate and butyrate) were measured in the present study. Individual SCFAs exert distinct biological functions and may have differential pro-inflammatory or anti-inflammatory effects (Cuciniello et al., 2023; Mann et al., 2024; Shin et al., 2023). Therefore, the association between CFUs of *Lactobacillus* and SCFA levels in the present study should be interpreted cautiously. Future studies will be necessary to clarify which SCFA component is most relevant to POCD.

### 5.6 Conclusion

In conclusion, the preventive effect of ulinastatin on cognitive decline after general anaesthesia was associated with control of gut dysbiosis, specifically through prevention of *Lactobacillus* decline, as reflected by changes in SCFAs in the gut, blood, and brain, and by altered Nrf2 expression in the gut and brain. Collectively, these findings highlighted the gut–brain axis as a clinically relevant therapeutic target and supported microbiome-targeted interventions as a promising strategy for preventing POCD in clinical practice.

## CRediT authorship contribution statement

**Eun-Hwa Cho:** Data curation, Formal analysis, Investigation, Methodology, Validation, Visualization, Writing – original draft. **Seung-Wan Hong:** Data curation, Formal analysis, Writing – original draft. **Eun-Hye Seo:** Data curation, Formal analysis, Supervision, Writing – original draft. **Seong-Hyop Kim:** Conceptualization, Data curation, Formal analysis, Funding acquisition, Methodology, Supervision, Validation, Visualization, Writing – original draft.

## Ethics declaration

### Animal subject

This study was conducted in accordance with the ARRIVE (Animal Research: Reporting of In Vivo Experiments) guidelines. This study was approved by the Institutional Animal Care and Use Committee (IACUC) of Konkuk University.

## Declaration of generative artificial intelligence (AI) use

During the preparation of the study, author did not use any generative AI.

## Financial support

The study was also supported by Hanlim Pharmaceutical Co., Ltd. (Seoul, Korea). The study was also supported National Research Foundation of Korea (NRF) grant funded by the Korea government (RS-2026-25468651).

## Declaration of competing interests

The authors declare that they have no known competing financial interests or personal relationships that could have appeared to influence the work reported in this paper.

## Data Availability

Data will be made available on request.

## References

[bib1] Aghamohammad S., Hafezi A., Rohani M. (2023). Probiotics as functional foods: how probiotics can alleviate the symptoms of neurological disabilities. Biomed. Pharmacother..

[bib2] Ahmad Alwi N.A., Lim S.M., Mani V., Ramasamy K. (2023). Lactobacillus spp.-enhanced memory is strain-dependent and associated, in part, with amyloidogenic and anti-Oxidant/Oxidative stress interplay in amyloid beta precursor protein transgenic mice. J. Diet. Suppl..

[bib3] Arefayne N.R., Berhe Y.W., van Zundert A.A. (2023). Incidence and factors related to prolonged postoperative cognitive decline (POCD) in elderly patients following surgery and anaesthesia: a systematic review. J. Multidiscip. Healthc..

[bib4] Bairamian D., Sha S., Rolhion N., Sokol H., Dorothée G., Lemere C.A., Krantic S. (2022). Microbiota in neuroinflammation and synaptic dysfunction: a focus on Alzheimer’s disease. Mol. Neurodegener..

[bib5] Baizabal-Carvallo J.F., Alonso-Juarez M. (2020). The link between gut dysbiosis and neuroinflammation in Parkinson’s disease. Neuroscience.

[bib6] Chen K.-H., Lin H.-S., Li Y.-C., Sung P.-H., Chen Y.-L., Yin T.-C., Yip H.-K. (2022). Synergic effect of early administration of probiotics and adipose-derived mesenchymal stem cells on alleviating inflammation-induced chronic neuropathic pain in rodents. Int. J. Mol. Sci..

[bib7] Cheng J., Hu H., Ju Y., Liu J., Wang M., Liu B., Zhang Y. (2024). Gut microbiota-derived short-chain fatty acids and depression: deep insight into biological mechanisms and potential applications. Gen. Psychiatry.

[bib8] Cho E.-H., In C.-B., Lee G.-W., Hong S.-W., Seo E.-H., Lee W.H., Kim S.-H. (2024). The preventive effect of urinary trypsin inhibitor on postoperative cognitive dysfunction, on the aspect of behavior, evaluated by Y-maze test, via modulation of microglial activity. Int. J. Mol. Sci..

[bib9] Cho E.-H., Seo E.-H., Hong S.-W., Kim S.-H. (2024). The preventive effect of ulinastatin on blood–brain barrier dysfunction in rats with postoperative cognitive dysfunction after general anaesthesia with isoflurane. Int. J. Mol. Sci..

[bib10] Cuciniello R., Di Meo F., Filosa S., Crispi S., Bergamo P. (2023). The antioxidant effect of dietary bioactives arises from the interplay between the physiology of the host and the gut microbiota: involvement of short-chain fatty acids. Antioxidants.

[bib11] Dong Y., Cui C. (2022). The role of short-chain fatty acids in central nervous system diseases. Mol. Cell. Biochem..

[bib12] Duan M., Liu F., Fu H., Feng S., Wang X., Wang T. (2021). Effect of ulinastatin on early postoperative cognitive dysfunction in elderly patients undergoing surgery: a systemic review and meta-analysis. Front. Neurosci..

[bib13] Erny D., Hrabě de Angelis A.L., Jaitin D., Wieghofer P., Staszewski O., David E., Keren-Shaul H., Mahlakoiv T., Jakobshagen K., Buch T. (2015). Host microbiota constantly control maturation and function of microglia in the CNS. Nat. Neurosci..

[bib14] Fan Q., Wu Y., Li M., An F., Yao L., Wang M., Wang X., Yuan J., Jiang K., Li W. (2021). Lactobacillus spp. create a protective micro-ecological environment through regulating the core fucosylation of vaginal epithelial cells against cervical cancer. Cell Death Dis..

[bib15] Flores C.F.Y., Pineda Á.d.l.M.H., Bonilla V.M.C., Sáenz-Flor K. (2020). Sample management: stability of plasma and serum on different storage conditions. EJIFCC.

[bib16] Fock E., Parnova R. (2023). Mechanisms of blood–brain barrier protection by microbiota-derived short-chain fatty acids. Cells.

[bib17] Gao H., Li X., Chen X., Hai D., Wei C., Zhang L., Li P. (2022). The functional roles of Lactobacillus acidophilus in different physiological and pathological processes. J. Microbiol. Biotechnol..

[bib18] George M., Tharakan M., Culberson J., Reddy A.P., Reddy P.H. (2022). Role of Nrf2 in aging, Alzheimer's and other neurodegenerative diseases. Ageing Res. Rev..

[bib19] González-Bosch C., Boorman E., Zunszain P.A., Mann G.E. (2021). Short-chain fatty acids as modulators of redox signaling in health and disease. Redox Biol..

[bib20] Hey-Hadavi J., Velisetty P., Mhatre S. (2023). Trends and recent developments in pharmacotherapy of acute pancreatitis. PGM (Postgrad. Med.).

[bib21] Ibrahim F., Lebeer S., Salvetti E., Felis G.E. (2024). The genus lactobacillus—across the past and future. Lactic Acid Bacteria.

[bib22] Johnson S.L., Kirk R.D., DaSilva N.A., Ma H., Seeram N.P., Bertin M.J. (2019). Polyphenol microbial metabolites exhibit gut and blood–brain barrier permeability and protect murine microglia against LPS-Induced inflammation. Metabolites.

[bib23] Kim C.-S., Jung S., Hwang G.-S., Shin D.-M. (2023). Gut microbiota indole-3-propionic acid mediates neuroprotective effect of probiotic consumption in healthy elderly: a randomized, double-blind, placebo-controlled, multicenter trial and in vitro study. Clin. Nutr..

[bib24] Kim J., Kang H., Lee Y.-B., Lee B., Lee D. (2023). A quantitative analysis of spontaneous alternation behaviors on a Y-maze reveals adverse effects of acute social isolation on spatial working memory. Sci. Rep..

[bib25] Kim Y.S., Sohn S.-H., Min T.J. (2023). Protective effect of ulinastatin on cognitive function after hypoxia. Neuromolecular Med..

[bib26] Li L., Meng F., Li D. (2023). Downregulation of Nrf2 in the hippocampus contributes to postoperative cognitive dysfunction in aged rats by sensitizing oxidative stress and neuroinflammation. Oxid. Med. Cell. Longev..

[bib27] Li M., Su S., Cai W., Cao J., Miao X., Zang W., Gao S., Xu Y., Yang J., Tao Y.-X. (2020). Differentially expressed genes in the brain of aging mice with cognitive alteration and depression-and anxiety-like behaviors. Front. Cell Dev. Biol..

[bib28] Li Z., Zhu Y., Kang Y., Qin S., Chai J. (2022). Neuroinflammation as the underlying mechanism of postoperative cognitive dysfunction and therapeutic strategies. Front. Cell. Neurosci..

[bib29] Liang Z., Xu X., Qi X., Zhang F. (2021). Efficacy and safety of ulinastatin on cognitive dysfunction after general anesthesia in elderly patients: a protocol for systematic review and meta-analysis. Medicine.

[bib30] Liu B., Huang D., Guo Y., Sun X., Chen C., Zhai X., Jin X., Zhu H., Li P., Yu W. (2022). Recent advances and perspectives of postoperative neurological disorders in the elderly surgical patients. CNS Neurosci. Ther..

[bib31] Liu L., Huh J.R., Shah K. (2022). Microbiota and the gut-brain-axis: implications for new therapeutic design in the CNS. EBioMedicine.

[bib32] Liu Y., Tang T., Cai H., Liu Z. (2025). Bidirectional communication between the gut microbiota and the central nervous system. Neural Regen. Res..

[bib33] Lv Y., Meng Y., Rui H., Li T., Zhang J., Liu J., Ma X., Wang J., Yuan Y., Jiang Y. (2026). Ulinastatin attenuates capillary leakage and suppresses FoxO1-dependent angiopoietin-2 in sepsis-associated acute lung injury via PI3K pathway. PLoS One.

[bib34] Ma Y., Liu T., Fu J., Fu S., Hu C., Sun B., Fan X., Zhu J. (2019). Lactobacillus acidophilus exerts neuroprotective effects in mice with traumatic brain injury. J. Nutr..

[bib35] Mann E.R., Lam Y.K., Uhlig H.H. (2024). Short-chain fatty acids: linking diet, the microbiome and immunity. Nat. Rev. Immunol..

[bib36] Martin-Gallausiaux C., Marinelli L., Blottière H.M., Larraufie P., Lapaque N. (2021). SCFA: mechanisms and functional importance in the gut. Proc. Nutr. Soc..

[bib37] Meng Y., Qiu X., Tang Z., Mao Y., Tan Y. (2023). Lactobacillus paracasei L9 affects disease progression in experimental autoimmune neuritis by regulating intestinal flora structure and arginine metabolism. J. Neuroinflammation.

[bib38] Michael D., Jack A., Masetti G., Davies T., Loxley K., Kerry-Smith J., Plummer J., Marchesi J., Mullish B., McDonald J. (2020). A randomised controlled study shows supplementation of overweight and obese adults with lactobacilli and bifidobacteria reduces bodyweight and improves well-being. Sci. Rep..

[bib39] Oroojzadeh P., Bostanabad S.Y., Lotfi H. (2022). Psychobiotics: the influence of gut microbiota on the gut-brain axis in neurological disorders. J. Mol. Neurosci..

[bib40] Piao L., Na O.-H., Seo E.-H., Hong S.-W., Sohn K.-M., Kwon Y., Lee S.-H., Kim S.-H. (2022). Effects of general anaesthesia with an inhalational anaesthetic agent on the expression of exosomes in rats. Int. J. Med. Sci..

[bib41] Qin D., Ma Y., Wang Y., Hou X., Yu L. (2022). Contribution of lactobacilli on intestinal mucosal barrier and diseases: perspectives and challenges of Lactobacillus casei. Life.

[bib42] Qu Z., Sun J., Zhang W., Yu J., Zhuang C. (2020). Transcription factor NRF2 as a promising therapeutic target for Alzheimer's disease. Free Radic. Biol. Med..

[bib43] Sauer M., Han N.S. (2021). Lactic acid bacteria: little helpers for many human tasks. Essays Biochem..

[bib44] Seo E.-H., Piao L., Cho E.-H., Hong S.-W., Kim S.-H. (2023). The effect of ketamine on endoplasmic reticulum stress in rats with neuropathic pain. Int. J. Mol. Sci..

[bib45] Shin Y., Han S., Kwon J., Ju S., Choi T.G., Kang I., Kim S.S. (2023). Roles of short-chain fatty acids in inflammatory bowel disease. Nutrients.

[bib46] Silva Y.P., Bernardi A., Frozza R.L. (2020). The role of short-chain fatty acids from gut microbiota in gut-brain communication. Front. Endocrinol..

[bib47] Sugita S., Tahir P., Kinjo S. (2023). The effects of microbiome-targeted therapy on cognitive impairment and postoperative cognitive dysfunction—A systematic review. PLoS One.

[bib48] Tang H., Huang W., Yao Y.-F. (2023). The metabolites of lactic acid bacteria: classification, biosynthesis and modulation of gut microbiota. Microb. Cell.

[bib49] Tong C., Halengbieke A., Cao T. (2024). Efficacy and safety of ulinastatin in the treatment of septic shock: a systematic review and meta-analysis. Med. Data Min..

[bib50] Wang G., Zhu G., Chen C., Zheng Y., Ma F., Zhao J., Lee Y.-K., Zhang H., Chen W. (2021). Lactobacillus strains derived from human gut ameliorate metabolic disorders via modulation of gut microbiota composition and short-chain fatty acids metabolism. Benef. Microbes.

[bib51] Wang X.-q., Li H., Li X.-n., Yuan C.-h., Zhao H. (2021). Gut-brain axis: possible role of gut microbiota in perioperative neurocognitive disorders. Front. Aging Neurosci..

[bib52] Winter S.E., Bäumler A.J. (2023).

[bib53] Xu L., Kasprzyk-Hordern B. (2023). Assessment of the stability of antimicrobials and resistance genes during short-and long-term storage condition: accounting for uncertainties in bioanalytical workflows. Anal. Bioanal. Chem..

[bib54] Yang X.-x., Yang R., Zhang F. (2022). Role of Nrf2 in Parkinson's disease: toward new perspectives. Front. Pharmacol..

[bib55] Yang Y., Liu Y., Zhu J., Song S., Huang Y., Zhang W., Hao J., Yang X., Gao Q., Ma Z. (2022). Neuroinflammation-mediated mitochondrial dysregulation involved in postoperative cognitive dysfunction. Free Radic. Biol. Med..

[bib56] Yin Z., Song R., Yu T., Fu Y., Ding Y., Nie H. (2025). Natural compounds regulate macrophage polarization and alleviate inflammation against ALI/ARDS. Biomolecules.

[bib57] Zhao Q., Wan H., Pan H., Xu Y. (2024). Postoperative cognitive dysfunction—current research progress. Front. Behav. Neurosci..

