# Supplementary material for: Preventive effect of ulinastatin on postoperative cognitive dysfunction through modulation of the gut microbiome: evidence from short-chain fatty acids
Source: Brain Behav Immun Health. 2026 Aug 12;56:101327. doi: 10.1016/j.bbih.2026.101327 (PMC13499204; doi:10.1016/j.bbih.2026.101327)
Supplement: Supplementary file 1 [file mmc1.docx]

***Immunofluorescence staining for Nrf2 in gut and brain***

The stored colon samples in 15-mL conical tubes were transferred to paraffin cassettes. The cassettes containing the tissue were washed in water for 1 hour to remove residual 4% PFA. The washed cassettes were then placed in a tissue processor (Leica Biosystems, Germany) for paraffin processing. The programme was set as follows: formalin 1 for 1 hour, formalin 2 for 1 hour, 70% ethanol for 1 hour, 80% ethanol for 1 hour, 90% ethanol for 1 hour, 100% ethanol 1 for 1 hour, 100% ethanol 2 for 1 hour, xylene 1 for 1 hour, xylene 2 for 1 hour, paraffin 1 for 2 hours, and paraffin 2 for 2 hours.

After processing, the cassettes were transferred to a tissue embedding centre (Leica Biosystems, Germany) for paraffin embedding. Following embedding, the cassettes were placed in a freezer for 30 minutes to solidify the tissue. Tissue sections were then cut using a microtome (Leica Biosystems, Germany) and floated in a warm water bath at 37°C. Sections were mounted onto microscope slides and dried on a hotplate for 1 hour at 40°C and an additional 30 minutes at 56°C.

The slides were immersed in xylene 1 for 15 minutes and xylene 2 for 10 minutes to remove paraffin. The tissue was then rehydrated through graded ethanol: 100% ethanol 1 for 3 minutes, 100% ethanol 2 for 3 minutes, 90% ethanol for 3 minutes, 80% ethanol for 3 minutes, and 70% ethanol for 3 minutes. For antigen retrieval, the slides were immersed in 1× citrate buffer and microwaved for 5 minutes, repeated three times. The slides were then cooled for 20 minutes at room temperature and incubated with 5% goat serum (Abcam, UK; Cat# ab7841) as a blocking solution.

After blocking, the slides were washed five times with 1× PBS. Nrf2 antibody (Invitrogen, USA; Cat# PA5-27882) was used as the primary antibody, diluted 1:1,000 in 5% goat serum. The slides were incubated with the primary antibody for 1 hour at room temperature and then washed five times with 1× PBS. A 488 anti-rabbit secondary antibody (Invitrogen, USA; Cat# A-11008), diluted 1:1,000 in 5% goat serum, was applied and incubated in the dark for 1 hour at room temperature. The slides were again washed five times with 1× PBS.

For nuclear staining, 4′,6-diamidino-2-phenylindole (DAPI) (Invitrogen, USA; Cat# P36931) was diluted 1:2,000 in distilled water and applied to the slides for 5 minutes in the dark. After washing five times with 1× PBS, the slides were dehydrated through 70%, 80%, and 90% ethanol (3 minutes each), followed by 100% ethanol 1 and 100% ethanol 2 (3 minutes each), and xylene 1 and xylene 2 (3 minutes each).

After completion of staining, the sections were mounted with a coverslip using Anti-fade mounting medium (Vector, USA; Cat# H-1000). The coverslip was sealed with nail polish to prevent drying and movement. The stained sections were examined using a fluorescence microscope (Olympus, Japan).

The stored left hemisphere was processed using the same method as described for the colon samples.

***Western blot for Nrf2 in gut and brain***

The stored colon samples in microtubes were homogenised and centrifuged at 12,000 × *g* for 15 minutes at 4°C. After centrifugation, the supernatant was transferred to a new microtube and used for western blot analysis. Protein concentration was quantified using a bicinchoninic acid protein assay kit (Thermo, USA; Cat# 23225).

A total of 30 μg protein was prepared in a microtube by mixing the supernatant with 5× sodium dodecyl sulphate–polyacrylamide gel electrophoresis (SDS-PAGE) sample loading buffer (NZYTech, Portugal; Cat# MB10101) and distilled water. The mixture was vortexed and briefly spun down, then heated at 100°C for 5 minutes to denature the proteins. After heating, the tube was cooled on ice for 3 minutes, vortexed again, and centrifuged at 12,000 × *g* for 5 minutes at 4°C.

1× running buffer (All for Lab, Korea; Cat# AFL026) was added to the electrophoresis chamber (Bio-Rad, USA), and 10% mini SDS-PAGE gels (Bio-Rad, USA; Cat# 4561033) were placed in the chamber. The prepared samples and Pageruler™ prestained protein ladder (Thermo, USA; Cat# 26616) were loaded onto the gels. The chamber lid was closed, and electrophoresis was performed using a PowerPac HC power supply (Bio-Rad, USA) at 60 V for 30 minutes for the stacking gel, followed by 120 V for 2 hours for the running gel.

After electrophoresis, cold 1× transfer buffer (All for Lab, Korea; Cat# AFL028) was added to the chamber. The gels were removed and equilibrated in 1× transfer buffer (Deajung, Korea; Cat# 7531-4400). Proteins were transferred onto Immuno-Blot^®^ polyvinylidene difluoride membranes (Bio-Rad, USA; Cat# 1620174) in transfer buffer using the PowerPac HC power supply at 80 V for 150 minutes.

Following transfer, the membranes were washed with 1× tris-buffered saline with Tween 20 (TBS-T) (All for Lab, Korea; Cat# AFL013) for 15 minutes, 3 times. The membranes were then blocked with 5% bovine serum albumin (BSA) (Santa Cruz Biotechnology, USA; Cat# sc-2323) for 2 hours at room temperature. After blocking, the membranes were washed again with 1× TBS-T for 15 minutes, 3 times.

To assess Nrf2 expression, membranes were incubated with primary Nrf2 antibody diluted 1:1,000 in 5% BSA for 1 hour at room temperature, followed by overnight incubation at 4°C. The next day, membranes were washed with 1× TBS-T for 15 minutes, 3 times. Goat anti-rabbit immunoglobulin G (H+L) horseradish peroxidase–conjugated secondary antibody (GenDEPOT, USA; Cat# SA002), diluted 1:5,000 in 5% BSA, was applied for 1 hour at room temperature. After incubation, the membranes were washed again with 1× TBS-T for 15 minutes, 3 times.

Finally, the membranes were treated with Pierce™ enhanced chemiluminescence (ECL) western blotting substrate (Thermo, USA; Cat# 32209), and protein expression was detected using the iBright CL1000 imaging system (Thermo, USA).

The stored right hemisphere samples were analysed using the same method as described for the colon samples.
